# One-piece bifrontal basal craniotomy with pericranial flap for frontal sinus management: How I Do It

**DOI:** 10.1007/s00701-025-06528-1

**Published:** 2025-04-17

**Authors:** Eiji Ito, Ryotaro Sugita, Mao Yokota, Tadashi Watanabe

**Affiliations:** 1 Skull Base Surgical Center, Aichi Medical University Hospital, 1 - 1 Yazakokarimata, Nagakute, Aichi 480 - 1195 Japan; 2 Department of Neurosurgery, Gifu Prefectural Tajimi Hospital, Tajimi, Japan; 3 Department of Neurosurgery, Aichi Medical University, Nagakute, Japan

**Keywords:** One-piece craniotomy, Pericranial flap, Anterior skull base surgery, Frontal sinus reconstruction, Cerebrospinal fluid leakage

## Abstract

**Background:**

Anterior skull base surgery poses challenges including cerebrospinal fluid (CSF) leakage and cosmetic concerns. A novel one-piece bifrontal basal craniotomy using a pericranial flap was developed to improve outcomes.

**Method:**

The technique included precise burr hole placement, dura-protective craniotomy, and frontal sinus sealing using a pericranial flap. Reconstruction was stabilized using miniplates and screws, leaving the epidural dead space unfilled and lowering infection risks.

**Conclusion:**

This method reduces CSF leakage, enhances reconstruction durability, and preserves cosmetic and functional outcomes, offering a reliable approach for anterior skull base surgery.

**Supplementary Information:**

The online version contains supplementary material available at 10.1007/s00701-025-06528-1.

## Relevant surgical anatomy

The frontal sinus (FS) in the frontal bone of the skull behind the brow ridges is paired with air-filled cavities. These paranasal sinuses are typically triangular between the external and internal plates of the frontal bone [1]. The average FS measures 45.8 ± 12.3 mm in breadth, 29.8 ± 7.3 mm in height, and 22.7 ± 4.8 mm in depth [2]. The average FS volume is 8.9 ± 6.3 cm^3^ in men and 5.2 ± 3.1 cm^3^ in women [2]. They extend superiorly into the squamous part of the frontal bone and posteriorly into the orbital part.

The nasofrontal duct, a funnel-shaped structure on the posterior medial floor of the FS, [3] drains into the middle meatus of the nasal cavity, maintaining sinus function. Variability in FS size, shape, and drainage pathways necessitates careful preoperative planning to prevent complications, such as cerebrospinal fluid (CSF) leakage, pneumocephalus, and infection, during anterior skull base procedures.

## Description of the technique

### One-piece bifrontal basal craniotomy

**Fig. 1:**
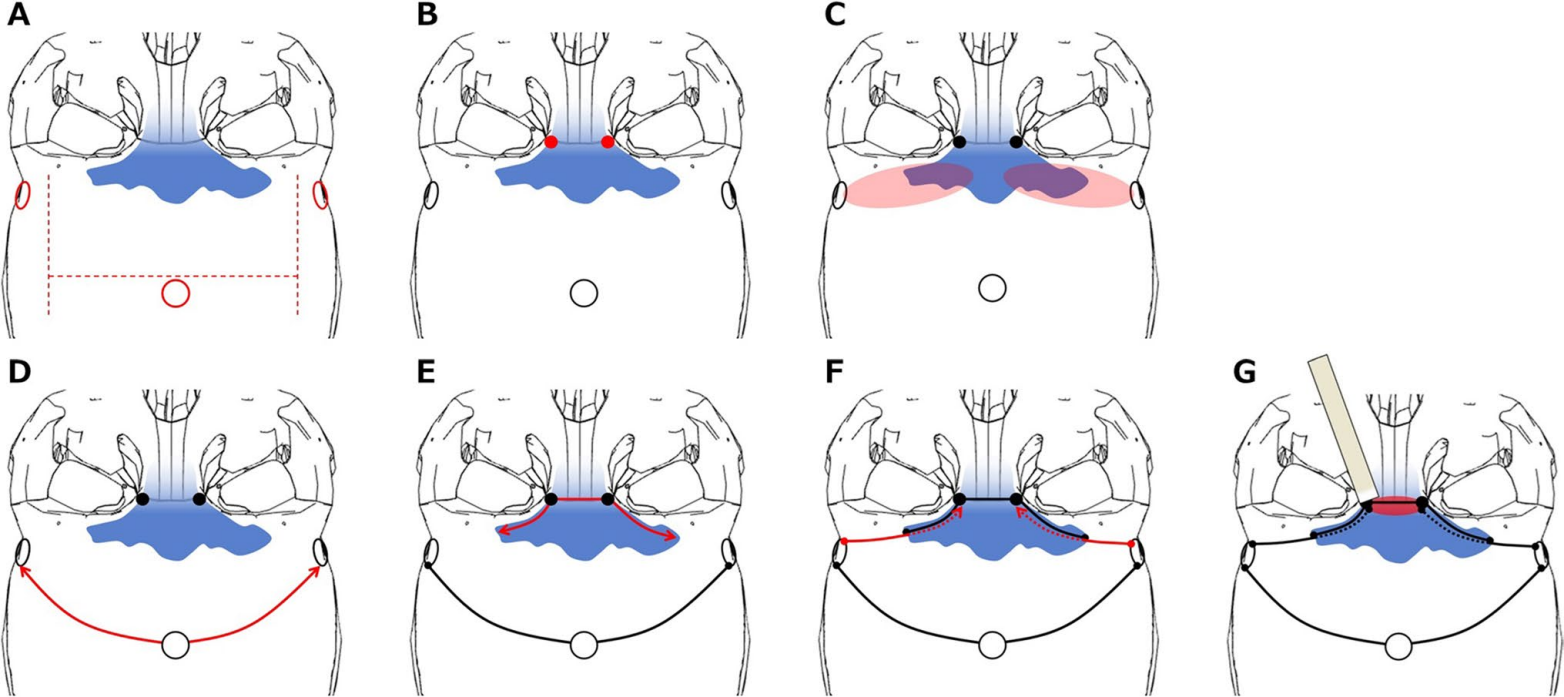
One-piece bifrontal basal craniotomy. **A** Schematic of “H”-shaped pericranial flap design (red ditto line). The midline burr hole above the superior sagittal sinus and bilateral frontobasal burr holes beneath the temporal muscles are used to avoid hollow deformities (red circle). **B** Small drill holes (red dot) at the superomedial orbital corners near the frontonasal suture as precise osteotomy entry points. **C** Pericranial stripper insertion through the lateral frontobasal burr holes for epidural dissection in the basal frontal area (red zone) to avoid dural lacerations. **D** Craniotomy (red line) technique connecting the superior sagittal sinus and bilateral frontobasal burr holes. **E** Outer frontal sinus (FS) plate incision from the superomedial orbital burr hole to the lateral boundary (red line), preserving structural integrity. **F** The inner FS plate is incised along the outer plate trajectory (red line) to protect the dura mater. **G** The inner wall of the FS and frontal septum is cut using a chisel (red zone), preserving the structural integrity of the inner plate for reconstruction

**Fig. 2:**
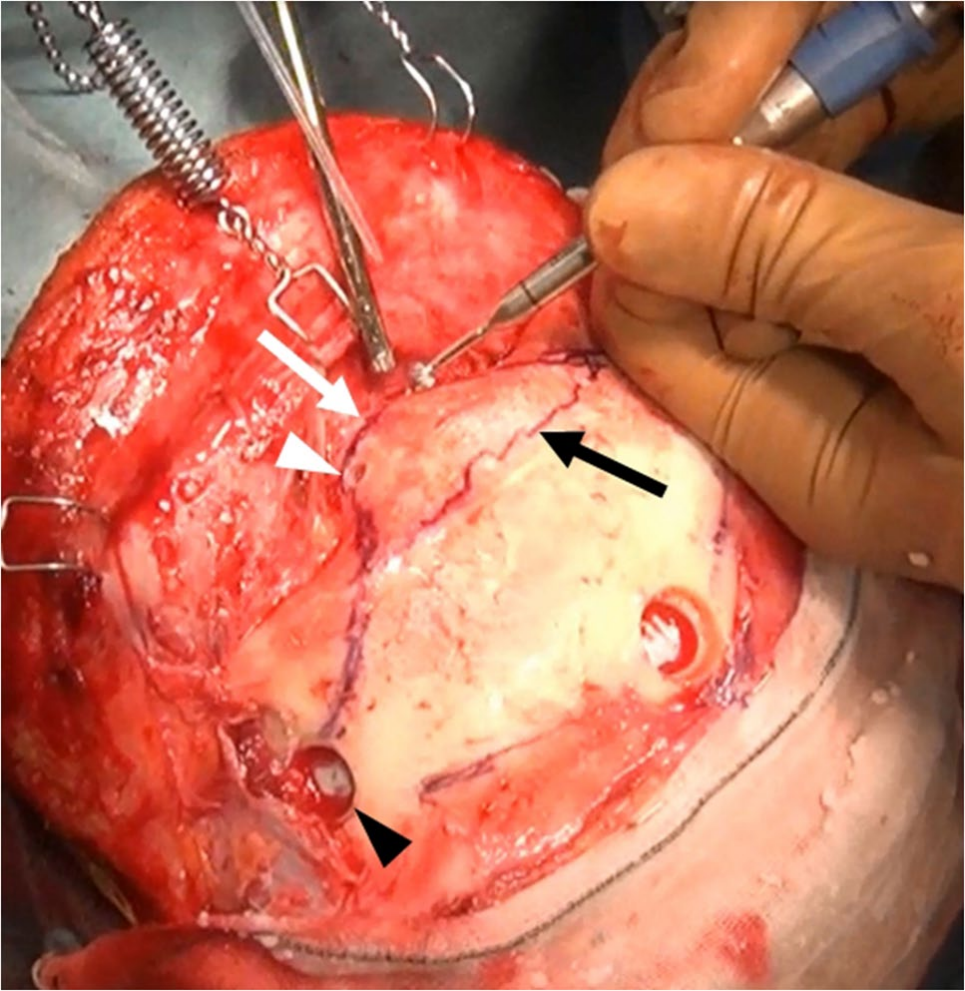
Intraoperative view showing drill holes for frontal sinus (FS) osteotomy. Supramedial orbital hole (white arrowhead) created at the frontonasal suture (white arrow) using a 3-mm cutting drill and lateral frontobasal burr hole (black arrowhead) under the temporal muscle. Marking of rostral FS margin (black arrow) using an intraoperative navigation system

**Fig. 3:**
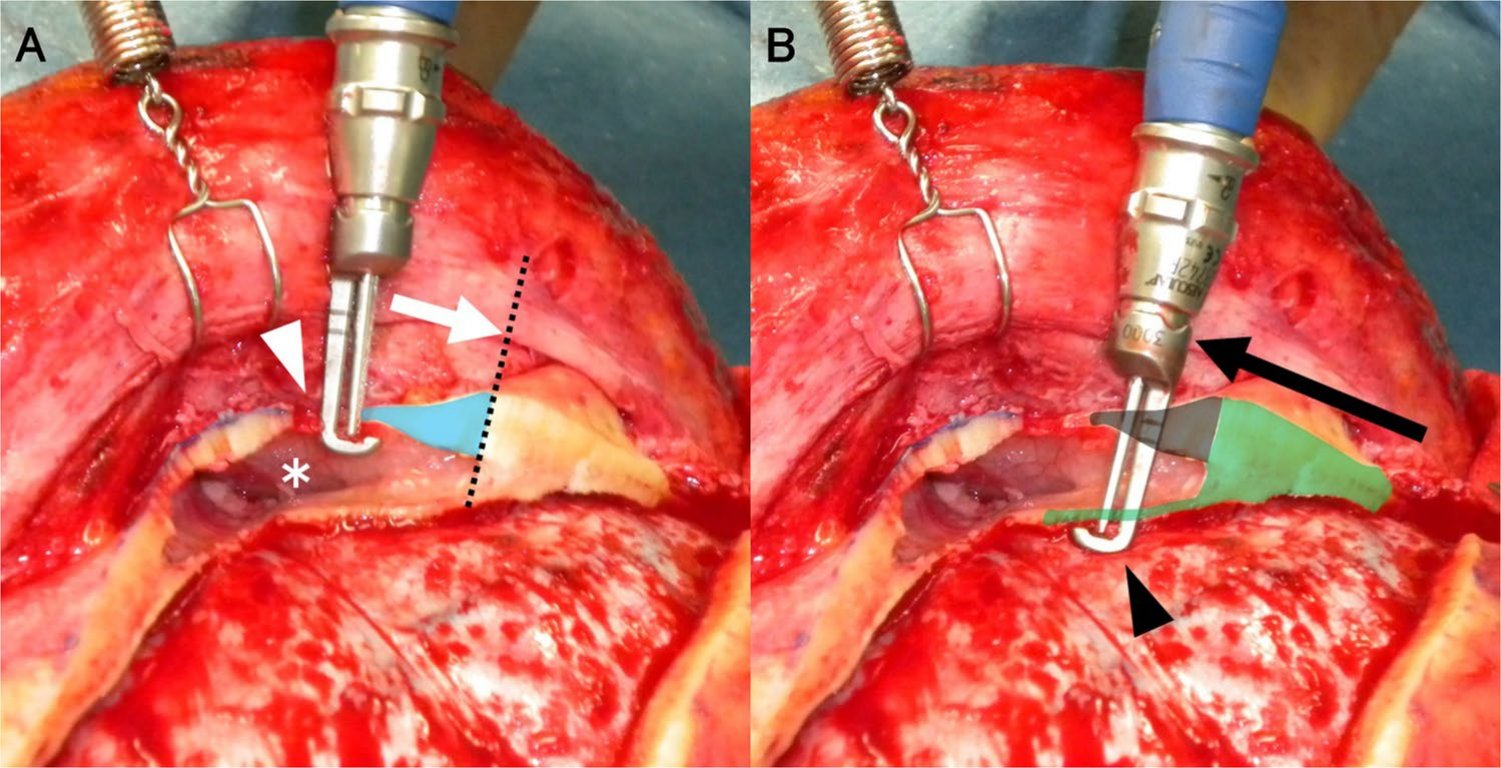
Cross-sectional diagram of osteotomized outer and inner frontal sinus (FS) plates. **A** The outer FS plate (light-blue zone) is cut using a craniotome with a dura guard (white arrow) from the supramedial orbital hole (white arrowhead) to the lateral FS limit (dotted line). FS is opened (white asterisks). **B** The inner FS plate is cut using a craniotome with a dura guard (black arrow head) from the lateral frontobasal burr hole to the ipsilateral superomedial orbital hole (black arrow, light green zone). The inner plate is cut through the previously created outer plate space (grey zone)

1. Pericranial Flap Creation
  Bilateral symmetrical skin incisions were made along the hairline to minimize scarring. The pericranial flap was designed in an “H” shape, [4] with the frontal base segment sealing the FS and the parietal segment stabilizing titanium plates (Fig. 1A). This preserves the temporal muscle integrity, reduces postoperative discomfort, and enhances structural stability.
2. Placement of Burr Holes
  A midline burr hole was made above the superior sagittal sinus to allow safe entry while protecting the sinus. Additional lateral frontobasal burr holes were drilled beneath the temporal muscles to prevent hollow deformities (Fig. 1A). Two small burr holes, 3 mm in diameter, were positioned at the superomedial orbital corners near the frontonasal suture to partially open the FS and provide precise osteotomy entry points (Figs. 1B and 2).
3. Epidural Dissection in the Basal Frontal Area
  The epidural layer was meticulously dissected using a pericranial stripper inserted through the lateral frontobasal burr holes to expose the basal frontal area (Fig. 1C). A craniotome with a dural guard was used to connect the burr holes, allowing controlled removal of the frontal bone (Fig. 1D).
4. Osteotomy of the Basal FS (Video 1)
  FS osteotomy was performed in two steps to preserve structural integrity. The outer FS plate was incised from the superomedial orbital burr hole to the lateral boundary (Figs. 1E and 3A). The inner plate was incised along the same trajectory, extending from each lateral frontobasal to the ipsilateral superomedial orbital burr hole (Figs. 1F and 3B). Special attention was paid to protect the dura mater. The frontonasal suture was excised and the inner FS wall and frontal septum were removed using a chisel, preserving the integrity of the inner plate for reconstruction (Fig. 1G).

### Reconstruction and closure (Video 2)

**Fig. 4:**
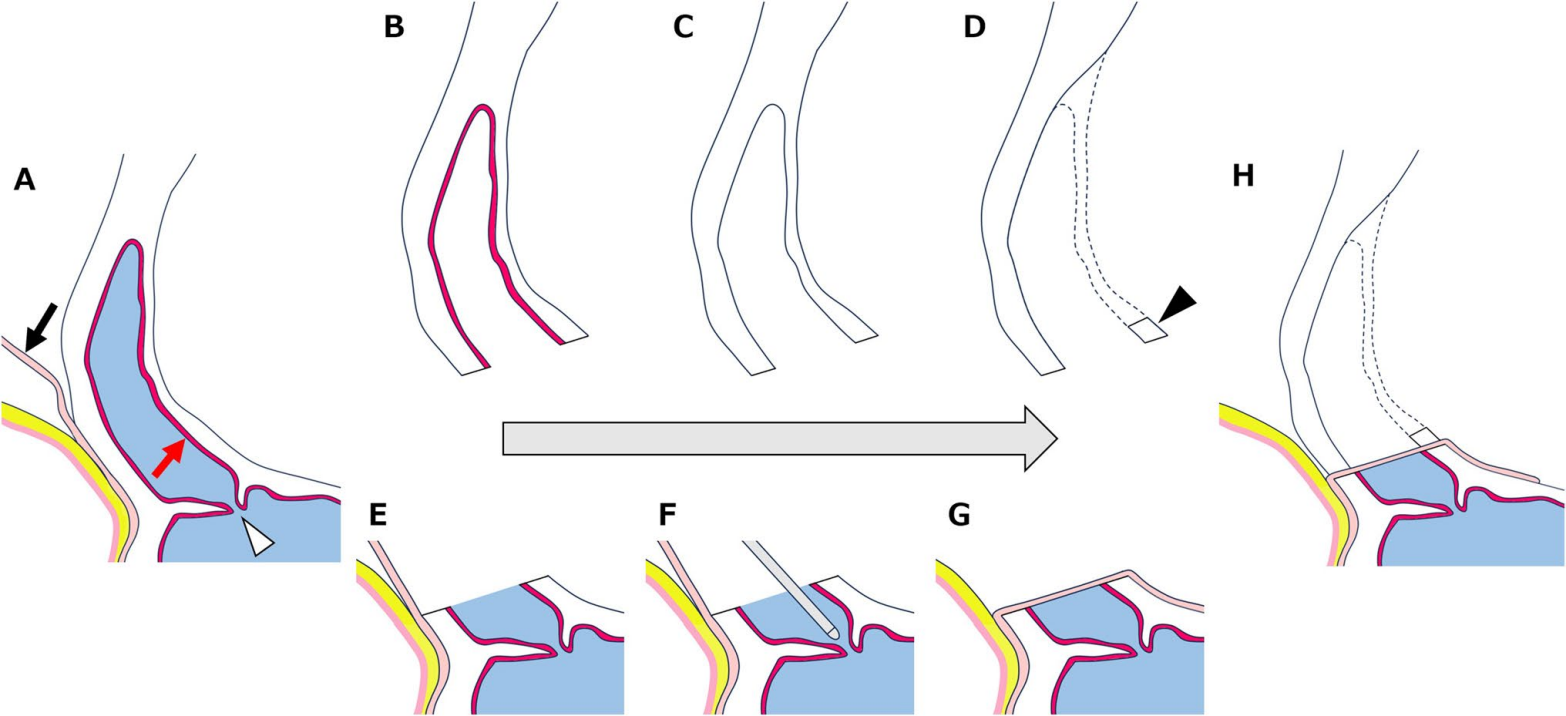
A step-by-step illustration of the reconstruction process. **A** Sagittal image of the anterior skull base after pericranial flap creation (black arrow). Red arrow: mucosa of frontal sinus, white arrowhead; nasofrontal duct. **B** One-piece bone flap with frontal sinus mucosa. **C** Frontal sinus mucosa removed completely. **D** Inner FS wall modification: partially excising the inner FS wall to create a stable reconstruction base. Black arrowhead: the basal portion of inner FS wall as a straight bar-like structure. **E** preservation of the mucosa tightly adherent to the FS sidewalls. **F** Nasofrontal duct inspection with probe: Ensuring duct patency to prevent dead space formation. **G** The superior wall of the FS is reconstructed using a pericranial flap. **H** Securing the pericranial flap between the skull base and cranial bone flap to reinforce closure

**Fig. 5:**
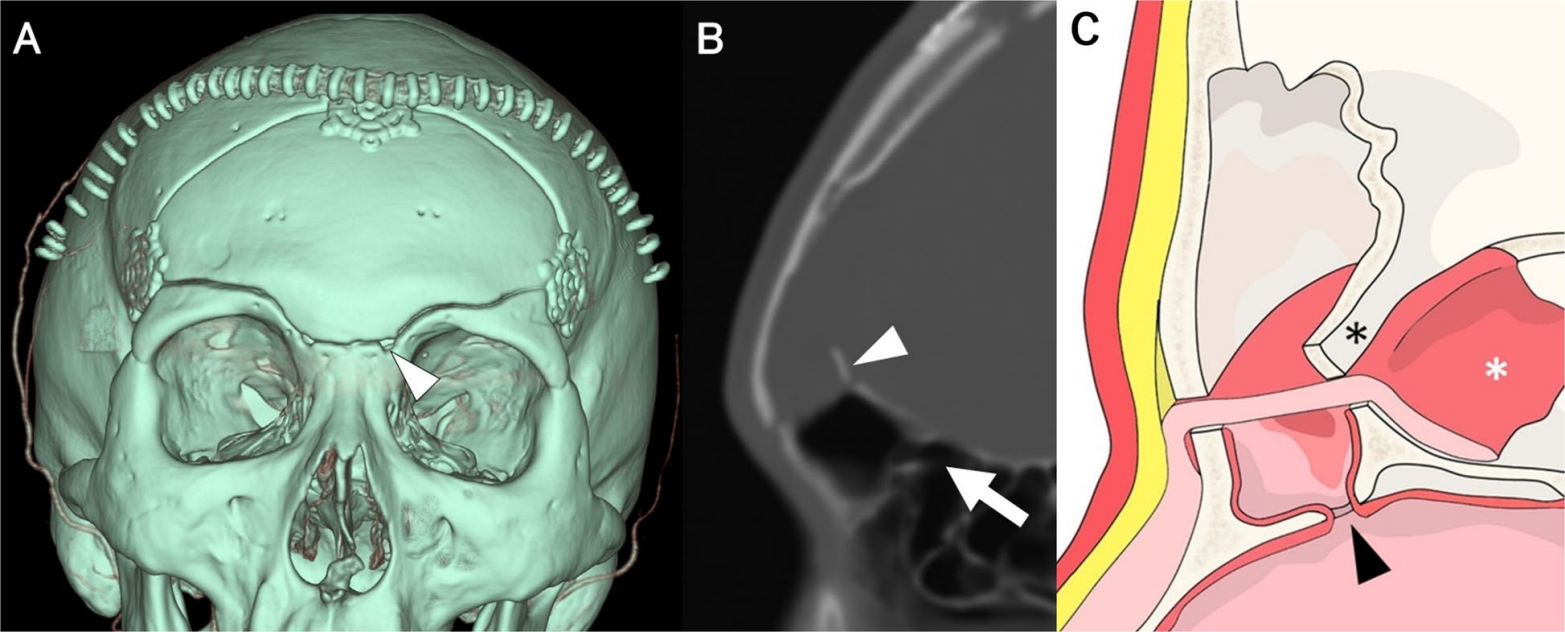
**A** Postoperative three-dimensional computed tomography (CT) reconstruction image showing a one-piece craniotomy with three titanium plates. White arrowhead: supramedial orbital hole. **B** Postoperative CT scan with a bone window showing a pericranial flap sandwiched on the edge of the frontal sinus stump between the skull base and bone flap on the reshaped inner walls (white arrowhead) and outer wall of the frontal sinus. White arrow indicates the patented nasofrontal duct. **C** Schematic illustration of frontal sinus reconstruction and closure. After confirming nasofrontal duct patency (black arrowhead) and preserving the side wall mucosa of frontal sinus, the orifice margin is covered by a pericranial flap (white asterisk). The flap is sandwiched between the skull base and cranialized reshaped bone flap. Black asterisk indicates the reshaped basal part of the inner wall of the FS bone flap resembling a straight bar

1. Nasofrontal Duct Inspection and Preservation of FS Mucosa
  During one-piece craniotomy, the superior wall of the FS is inevitably opened (Fig. 4B), and the mucosa in this region is removed completely (Fig. 4C). After dural closure, the nasofrontal duct was inspected, and if necessary, opened using a probe to restore patency (Fig. 4F). Therefore, the superior wall of FS is reconstructed using a pericranial flap (Fig. 4G). Unlike traditional methods where the entire FS mucosa is removed and the nasofrontal duct is occluded, our technique preserves the mucosa tightly adherent to the FS sidewalls (Fig. 4E). Maintaining nasofrontal duct patency is critical to prevent the formation of dead space and reduce the risk of postoperative infection. If the duct is not naturally open, it is intentionally opened using a probe to ensure adequate drainage.
2. Pericranial Flap Technique for FS Closure and Preservation of Frontalis Muscle Function
  A pericranial flap was securely attached to the frontobasal dura to protect and maintain the FS integrity. During cranialization, the inner wall was partially excised, leaving the basal portion as a straight bar-like structure (Figs. 4D and 5C). This design allows the pericranial flap to be securely sandwiched, enhancing closure integrity, and reducing CSF leakage risks. In addition, preserving frontalis muscle function also has cosmetic benefits.
3. Stabilization and Infection Prevention in Frontobasal Reconstruction
  The one-piece frontobasal bone flap was reattached using miniplates and screws for stabilization. The pericranial flap was positioned between the skull base and bone flap, creating a protective barrier (Figs. 4H and 5B). The epidural dead space was left unfilled to reduce infection risk and promote natural healing. Additionally, postoperative three-dimensional computed tomography (3D-CT) images (Fig. 5A) confirm the stability of the cranial reconstruction and the integrity of the pericranial flap placement.

### Postoperative management protocol: prophylactic antibiotics and early mobilization

Postoperatively, the patients received cefazolin sodium (2 g/d) for 2 d as prophylactic antibiotic treatment. Patients were advised to maintain head elevation at a 20° angle for 24 h postoperatively, after which activity restrictions were lifted to encourage mobilization. Spinal drainage was not performed in any patient.

## Indications

One-piece bifrontal basal craniotomy is indicated for extensive anterior skull base exposure, particularly in cases involving the FS. The technique is well-suited for pathologies such as meningiomas, craniopharyngiomas, and aneurysms, where precise FS management is critical for minimizing complications. Moreover, it offers robust cranial reconstruction and preserves both cosmetic and functional outcomes, making it a reliable option for complex cases.

## Limitations

This technique has limitations, particularly in cases of highly developed FS, where the distance between the outer and inner plates may hinder effective craniotomy owing to limited tool reach. Active FS infections, such as meningitis, are contraindicated because of the increased risk of postoperative complications. Hence, comprehensive preoperative assessment is crucial for ensuring feasibility and safety.

## How to avoid complications

To minimize complications, meticulous dural dissection is required around the superior sagittal sinus and FS. Preserving the FS mucosa and ensuring nasofrontal duct patency are critical in preventing mucocele formation. Leaving the inner FS plate as a straight bar-like structure allows the pericranial flap to be securely sandwiched, enhancing closure integrity, and reducing CSF leakage risks.

## Specific information for the patient

This technique offers multiple benefits, including a reduced risk of CSF leakage, infection, and mucocele formation. The pericranial flap preserves the frontalis muscle function, thus maintaining natural facial expressions. The elimination of postoperative spinal drainage enables earlier mobilization and quicker recovery. However, this procedure is not suitable for patients with a thick FS or active infections.

## Supplementary Information

Below is the link to the electronic supplementary material.

Video 1. One-piece Bifrontal Basal Craniotomy: Step-by-Step Surgical Technique. This video demonstrates the step-by-step procedure for performing a one-piece bifrontal basal craniotomy. The key steps include burr hole placement, epidural dissection, osteotomy of the frontal sinus, and controlled removal of the frontal bone flap. (MPG 408600 KB)

Video 2. Frontal Sinus Repair with Pericranial Flap and Bone Flap Fixation. This video demonstrates the repair of the frontal sinus following bifrontal basal craniotomy. The mucosa on the sidewall of the frontal sinus is preserved, and patency of the nasofrontal duct is confirmed. A pericranial flap is then placed to close the frontal sinus, its edge anchored to the anterior skull base dura. Finally, the bone flap, shaped with a straight bar-like structure at the basal inner plate, is used to sandwich the pericranial flap, ensuring secure fixation and stable reconstruction. (MPG 363870 KB)

## Data Availability

No datasets were generated or analysed during the current study.
